# The anti-osteoporotic effect of puerarin on the femoral bone in rat models of osteoporosis: a systematic review and meta-analysis

**DOI:** 10.3389/fphar.2025.1712682

**Published:** 2026-01-05

**Authors:** Zhaoxi Yang, Rui Tang, Yulin Ma, Wenlong Luo, Yimei Hu

**Affiliations:** The Clinical Medical College, Chengdu University of Chinese Medicine, Chengdu, Sichuan, China

**Keywords:** puerarin, osteoporosis, bone mineral density, meta-analysis, rats

## Abstract

**Objective:**

In this systematic review and meta-analysis, we aimed to evaluate the anti-osteoporotic efficacy of puerarin in rodent models of osteoporosis (OP) and to explore the impact of the dosage, treatment duration, and intervention method.

**Methods:**

A comprehensive search of electronic databases (e.g., PubMed, Embase, Web of Science, CNKI, and Wanfang) was conducted through August 2025. Randomized controlled trials investigating the effects of puerarin monotherapy on osteoporotic rats were included. The primary outcome measured was bone mineral density (BMD), and the secondary outcomes included bone histomorphometric parameters (BV/TV, Tb.Th, Tb.N, and Tb.Sp) and bone turnover markers (e.g., PINP, BALP, CTX, TRACP, and osteocalcin). Data were pooled using a random-effects model, and subgroup analyses were performed based on the puerarin dose, treatment duration, and intervention method. The study quality was assessed using the SYRCLE risk-of-bias tool.

**Results:**

Twenty-eight studies involving 570 animals were included. The meta-analysis demonstrated that puerarin significantly increased femoral BMD (SMD = 2.95, 95% CI: 2.32 to 3.58, and p < 0.00001) and improved the bone microarchitecture by increasing BV/TV, Tb.Th, and Tb.N, and decreasing Tb.Sp. Subgroup analysis revealed that the most pronounced BMD improvement occurred at doses ≥50 mg/kg/day administered for ≥8 weeks. Puerarin significantly suppressed bone resorption markers, CTX and TRACP, and elevated serum levels of osteocalcin, calcium, and phosphorus. However, its effects on bone formation markers, PINP and BALP, were not statistically significant.

**Conclusion:**

Puerarin exhibits significant therapeutic potential for OP in rat models by increasing BMD, improving bone quality, and rebalancing bone metabolism in favor of formation, primarily through the inhibition of resorption. The optimal effect appears to be dose- and duration-dependent. Although these preclinical findings are promising, the clinical translation of puerarin requires validation through larger-scale, high-quality animal studies and subsequent clinical trials.

## 1 Introduction

Osteoporosis (OP) is a systemic skeletal disease characterized by reduced bone mass, deterioration of bone microstructure, increased bone fragility, and elevated risk of fractures (Cosman et al., 2014). It represents a major global public health issue, affecting over 10 million Americans aged 50 years and above, a number that is expected to increase due to the aging population (Keshishi et al., 2021). In China, the prevalence of OP among adults aged 40 years or older is 5.0% in males and 20.6% in females (Wang et al., 2021). As its prevalence continues to increase with population aging, it imposes a substantial economic burden on society and has gained growing attention.

Treatment strategies for OP include dietary modifications, rehabilitative exercise, and pharmacological interventions. Among these, drug therapy is the most effective approach. However, calcium supplements and active vitamin D can only increase the bone calcium content and have limited effects on regulating the bone metabolic balance. Commonly used anti-osteoporotic medications are often associated with potential adverse effects; for example, denosumab frequently causes back pain and limb pain (Deeks, 2018), and teriparatide may impair the cardiovascular, central nervous, and endocrine systems, potentially inducing other systemic disorders (Lindsay et al., 2016). Therefore, exploring alternative treatments with improved safety profiles is of great importance.

Puerarin, an isoflavone monomer isolated and extracted from the dried roots of *Pueraria lobata*, has been demonstrated to possess multiple biological activities. Its impact on bone health has recently emerged as a new research focus. Puerarin has been confirmed to exhibit estrogen-like effects (Li and Yu, 2003; Wang et al., 2012). Data from *in vitro* and animal model studies indicate that puerarin can stimulate the expression of osteogenic markers such as bone alkaline phosphatase, type I collagen, osteoprotegerin, osteocalcin, and osteopontin (Li et al., 2016; Tiyasatkulkovit et al., 2012; Tiyasatkulkovit et al., 2014). Simultaneously, it inhibits osteoclast formation and the expression of bone resorption markers such as C-terminal telopeptide of type I collagen (CTX) (Guo et al., 2019). Thus, puerarin may be an effective compound for inhibiting bone resorption and improving the bone structure.

However, its clinical application remains limited due to insufficient clinical evidence. The current understanding of puerarin’s effects on bone tissue lacks a systematic evaluation of its efficacy. Meta-analyses of animal studies can help determine the efficacy and safety of pharmacological interventions. Therefore, it is necessary to conduct a systematic review to confirm the effectiveness of puerarin intervention. In this article, we perform a meta-analysis of relevant randomized controlled trials to evaluate the anti-osteoporotic effects of puerarin in osteoporotic rodent models, aiming to provide a reference for further clinical research.

## 2 Methods

The methodology of this study adheres to the preferred reporting items recommended by the guidelines for systematic reviews and meta-analyses (Liberati et al., 2009). The proposal has been registered in PROSPERO (registration number: CRD420251130437).

### 2.1 Literature retrieval

We conducted an electronic search of the following databases: Embase, PubMed, Web of Science, Cochrane Library, Chinese National Knowledge Infrastructure, and Wanfang. No time or language restrictions were set, and the retrieval date was August 2025. The search algorithm was adapted according to the different database requirements. For instance, the retrieval strategy for Web of Science was TS = [(puerarin OR kakonein OR “daidzein-8-C-glucoside”) AND (osteoporosis OR osteoporotic OR “bone loss” OR “bone density” OR BMD) AND (rat OR rats OR rodent OR rodents OR ovariectomized OR ORX OR OVX)].

### 2.2 Inclusion criteria

Animal studies that fulfilled the following conditions were included in our study:

1. Experimental groups received puerarin as monotherapy, whereas the corresponding control groups were treated with a vehicle or received a placebo such as saline solution.
2. Studies with conclusive results.
3. Animal models established using different methods, regardless of the species, age, weight, or gender.

### 2.3 Exclusion criteria

Studies with the following conditions were excluded from the analysis:

1. *In vitro* studies, case reports, clinical trials, reviews, abstracts, comments, and editorials.
2. Studies that did not use an acceptable established OP model.
3. Studies with missing data.
4. Duplicate publications.
5. Studies in which no outcome indicators were used.

### 2.4 Outcome measurements

Bone mineral density (BMD) was selected as the primary outcome for this meta-analysis as it represents the diagnostic gold standard for OP (Curry et al., 2018), and the secondary outcomes included the static parameters for the trabecular bone: namely, bone volume over total volume (BV/TV), trabecular number (Tb.N), trabecular thickness (Tb.Th), trabecular separation (Tb.Sp), serum osteocalcin (S-OCN), serum calcium (CA), serum phosphorus (P), C-terminal telopeptide of type I collagen (CTX-1), and procollagen type I N-terminal propeptide (PINP).

### 2.5 Selection of studies

After excluding the duplicate reports, we independently assessed the titles and abstracts of the remaining articles to exclude ineligible studies. Full texts of the remaining articles that met the inclusion criteria were reviewed to determine their eligibility for inclusion. Discrepancies in the selections between the authors were resolved through mutual discussion.

### 2.6 Risk-of-bias assessment

We independently evaluated the bias risk using the SYCLE tool (Hooijmans et al., 2014), comprising ten items across six domains: (1) selection bias, (2) performance bias, (3) detection bias, (4) attrition bias, (5) reporting bias, and (6) other bias. Studies meeting these criteria were considered low risk, whereas those not meeting them were deemed high risk. Studies with unclear bias descriptions were categorized as unclear risk. Any different opinions were resolved through mutual discussions.

### 2.7 Data extraction

Data were extracted by two authors independently and reviewed by a third author. The following information was extracted from each study: the author name, date of publication, animal species, age, sex, body weight, sample size, OP modeling methods, anesthetics method used, the intervention method for the control and experimental groups, and primary and secondary outcomes. We extracted the mean and standard deviation (SD) for the continuous variables.

### 2.8 Data analysis

The means and SD of the continuous variables were recorded in Microsoft Excel. Review Manager 5.3.0 and Stata 16.0 were used for data analysis and visualization. Heterogeneity among the studies was evaluated using the heterogeneity index, I^2^. When the heterogeneity was below 50%, a fixed-effects model was used; conversely, when heterogeneity exceeded the 50% threshold, a random-effects model was utilized. Subgroup and sensitivity analyses were carried out to identify the areas of variance, along with subgroup analyses by the type of investigation. *P* < 0.05 was considered statistically significant. When *I* ^2^ ≥ 50%, subgroup analysis was performed to probe for sources of dissimilarity. Sensitivity analysis was carried out using the leave-one-out approach to determine the robustness of outcome data. Publication bias was evaluated by visual inspection of funnel plots for asymmetry.

## 3 Results

### 3.1 Literature selection

**FIGURE 1:**
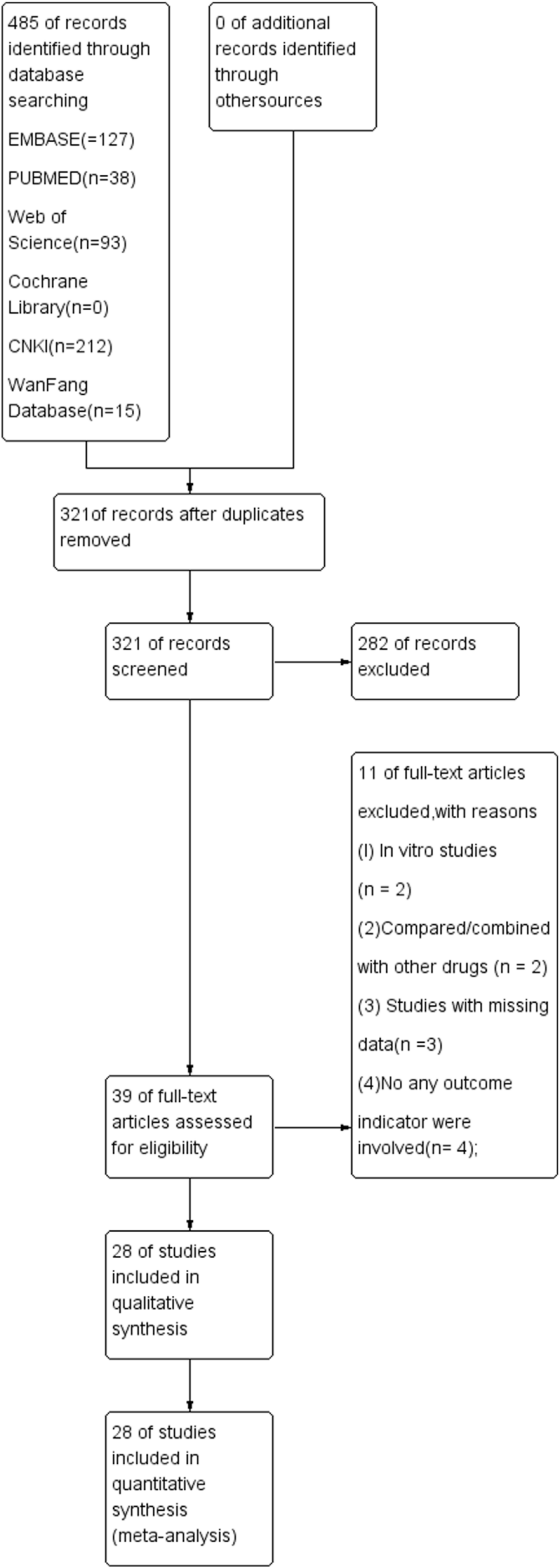
PRISMA flowchart of the study selection. PRISMA, preferred reporting items for systematic reviews and meta-analysis.

A total of 485 articles were identified after searching six databases, and 164 of them were excluded due to duplication. After reviewing the abstract, another 282 studies were eliminated. The remaining 39 studies were read in full, and 11 reports were excluded because of the following reasons: TS was compared/combined with other drugs, there was no control group, and/or there was duplication of data. Eventually, 28 studies were selected for this meta-analysis. The above selection process is shown in Figure 1.

### 3.2 Characteristics of the study

In this meta-analysis, we included a total of 28 articles, involving 28 studies and comprising bone density samples from 570 animals. The intervention consisted of puerarin administration, with a dosage range of 2 mg–200 mg/kg/day, primarily delivered via gavage, intraperitoneal injection, and subcutaneous injection. Outcome measures in the included studies comprised 28 assessments of femoral bone mineral density, 10 measurements of bone volume fraction (BV/TV), nine measurements of trabecular number (Tb.N), eight measurements of trabecular thickness (Tb.Th), and 11 measurements of trabecular separation (Tb.Sp), among others. The study duration ranged from 4 weeks to 112 days (Table1).

**TABLE 1:** Characteristics of the included studies.

First author (year)	Animals	Induction of osteoporosis	Age (weeks)	Weight	Treatment	Intervention (Ex)	Intervention (Con)	Sample size (Ex)	Sample size (Con)	Duration
Guo et al. (2019)	Male SD rats	STZ	7∼8	180 g–190 g	Gavage	PU 50 mg/kg.day	Standard chow and water	10	10	14 weeks
Li et al. (2020)	Female SD rats	OVX	10	——	Gavage	PU 100 mg/kg.day	Normal saline	10	10	14 weeks
Li et al. (2022)	Female SD rats	OVX	10	280 g–320 g	Gavage	PU 100 mg/kg.day	Normal water	10	10	14 weeks
Xiao et al. (2020)	C57BL/6J mice	OVX	11	——	IP	PU 100 mg/kg.2 day	Intraperitoneally with 1% DMSO in PBS	6	6	6 weeks
Wang et al. (2012)	Female SD rats	OVX	——	——	Gavage	PU 20 mg/kg.day	Distilled water	4	4	12 weeks
Yuan et al. (2016)	Female mice	OVX	8	——	Housing	PU 8 mg/kg.day	Normal saline	8	8	4 weeks
Tang et al. (2020)	Male SD rats	PIO	——	350 g	IP	PU 30.8 mg/kg.day	Normal saline	5	5	4 weeks
Yang et al. (2018)	Female SD rats	OVX	6	200 g	Gavage	PU 4 mg/kg.day	Normal saline	6	6	12 weeks
Qiu et al. (2022)	Female SD rats	OVX	24	230.6 ± 10.3 g	Gavage	PU 150 mg/kg.day	Normal saline	9	9	12 weeks
Wang et al. (2024)	Female SD rats	OVX	——	——	Gavage	PU 200 mg/kg.day	Normal saline	5	5	30 days
Zhang et al. (2024)	Female SD rats	OVX	24–32	280 g	SC	PU 35 mg/kg.day	Normal saline	15	15	6 weeks
Zhao et al. (2024)	Female SD rats	OVX	8	200 ± 20 g	SC	PU 80 mg/kg.day	Normal saline	8	8	8 weeks
Zhou et al. (2025)	Female SD rats	OVX	——	240 g–260 g	IP	PU 30 mg/kg.day	Normal saline	20	20	7 weeks
Yang et al. (2021)	Female SD rats	OVX	13	230 g–260 g	SC	PU 35 mg/kg.day	Normal saline	11	11	6 weeks
Wang and Lin (2019)	Female SD rats	OVX	13	225 g–258 g	Gavage	PU 50 mg/kg.day	Distilled water	10	10	10 weeks
Fang et al. (2021)	Female SD rats	OVX	20	——	SC	PU 50 mg/kg.day	Normal saline	8	8	12 weeks
Xu et al. (2021)	Female mice	OVX	12	(24 ± 2)g	Gavage	PU 100 mg/kg.day	Normal saline	10	10	8 weeks
Chen and Hu (2021)	Female Wistar rats	OVX	12	230 g–260 g	SC	PU 35 mg/kg.day	Normal saline	9	9	6 weeks
Niu et al. (2019)	Female SD rats	OVX	7∼8	260 g–320 g	SC	PU 50 mg/kg.day	Normal saline	14	14	8 weeks
Zeng et al. (2019)	Male SD rats	GIOP	——	220 g–240 g	IP	PU 200 mg/kg.day	Normal saline	10	10	8 weeks
Zeng and Shi (2018)	Female SD rats	OVX	——	220 g–240 g	Gavage	PU 100 mg/kg.day	Distilled water	10	10	3 months
Yu et al. (2019)	Female SD rats	OVX	20	180 g–220 g	SC	PU 40 mg/kg.day	Normal saline	10	10	112 days
Lyu and Fan (2023)	Female Wistar rats	OVX	——	180 ± 20 g	SC	PU 35 mg/kg.day	Normal saline	15	15	4 weeks
Li et al. (2019)	Female Wistar rats	HLS	8	160 g–180 g	Gavage	PU 15.4 mg/kg.day	Distilled water	10	10	4 weeks
Yue et al. (2021)	Female SD rats	OVX	16	320 g–350 g	SC	PU 35 mg/kg.day	Normal saline	12	12	6 weeks
Xi et al. (2018)	Female Wistar rats	——	4	109.45 g–119.44 g	Gavage	PU 15.4 mg/kg.day	Distilled water	10	10	12 weeks
Wang et al. (2020)	Female SD rats	OPF	12	350 ± 20 g	SC	PU 35 mg/kg.day	Normal saline	20	20	6 weeks
Yang and Guan (2023)	Female SD rats	OVX	——	200 g–257 g	IP	PU 30 mg/kg.day	Normal saline	10	10	6 weeks

IP, intraperitoneal injection; SC, subcutaneous injection.

### 3.3 Risk of bias

**FIGURE 2:**
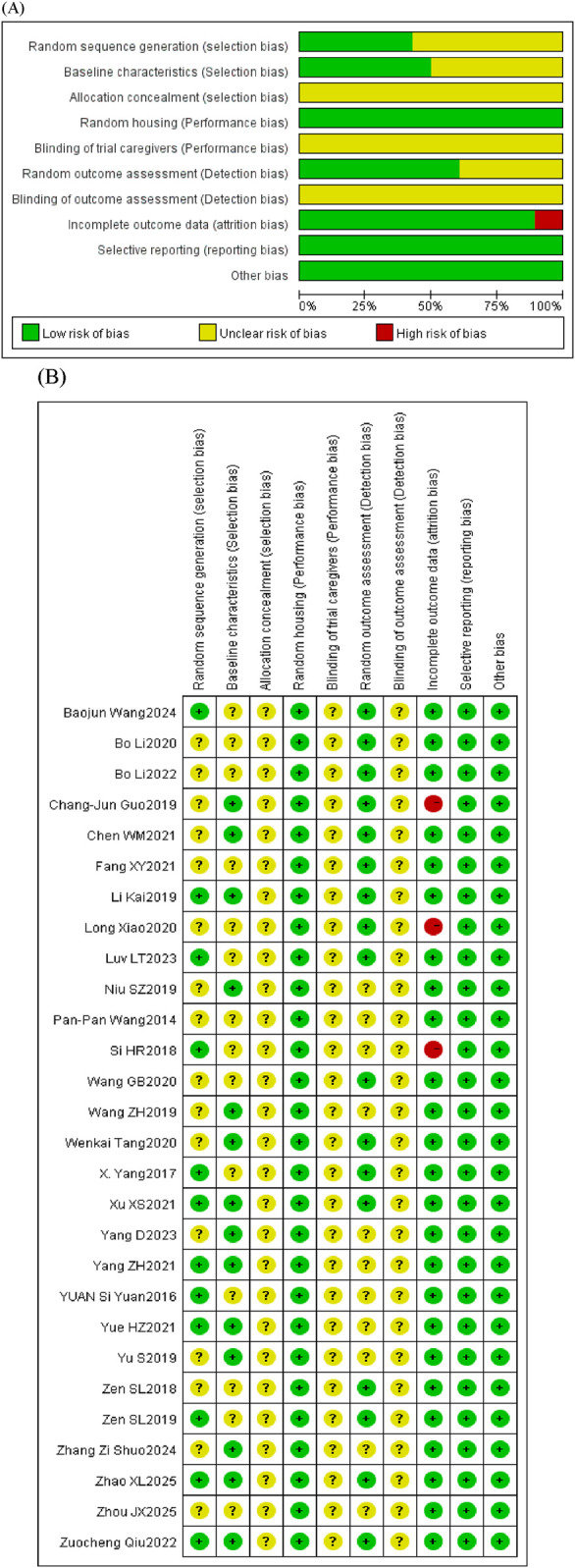
Risk of bias of the included studies. **(A)** Graph of the risk of bias. **(B)** Summary of the risk of bias.

The bias risk assessment results for the animal studies, as evaluated by the SYCLE tool, are summarized in Figures 2A,B; Supplementary Figure S1. The selection bias consists of “random sequence generation,” “baseline characteristics,” and “allocation concealment”; the performance bias consists of “random housing” and “blinding of trial caregivers”; the detection bias consists of “random outcome assessment” and “blinding of outcome assessment”; the attrition bias consists of “incomplete outcome data”; the reporting bias consists of “selective outcome reporting”; and other bias consists of “other sources of bias.”

None of the studies met all the methodological criteria that were evaluated. Regarding selection bias, 57.14% of the studies (n = 16) did not clearly describe the method of random sequence generation, whereas 50% (n = 14) did not clearly report baseline characteristics. Unclear risks of bias that were identified in the studies primarily involved allocation concealment, blinding of caregivers and/or investigators, and blinding of outcome assessors. A total of 39.28% of studies (n = 11) reported unclear methods for random outcome assessment, indicating detection bias. A total of 7.89% of studies (n = 3) were considered to have a high risk of bias due to incomplete outcome data.

### 3.4 Meta-analysis

#### 3.4.1 Bone mineral density

**FIGURE 3:**
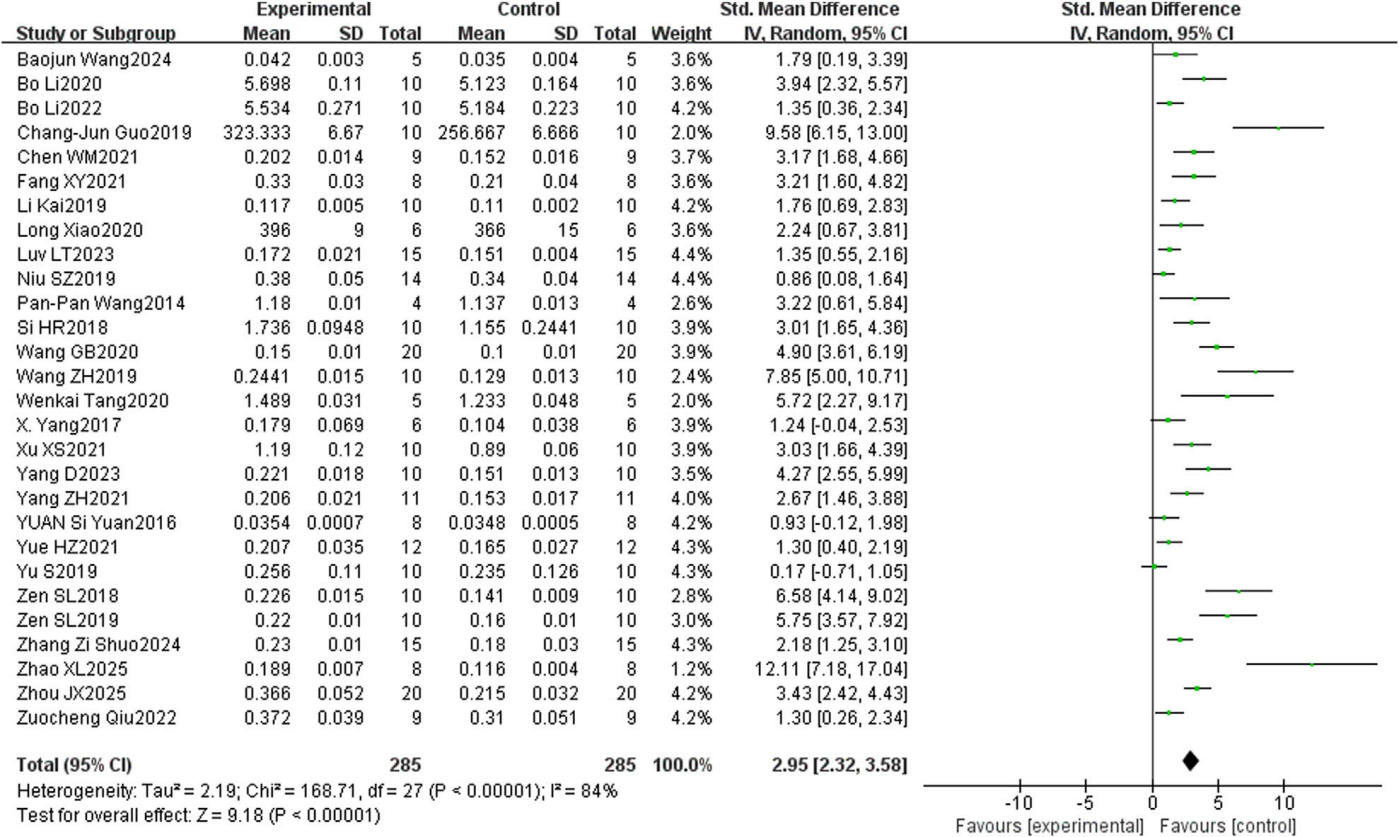
Forest plot comparing bone mineral density between the puerarin group and the control group. SD, standard deviation; std, standard.

The analysis on puerarin improving BMD in osteoporotic rats included 28 studies. The results showed that femoral BMD was significantly higher in the puerarin-treated group than in the control group (SMD = 2.95, 95% CI = 2.32 to 3.58, and p < 0.00001) (Figure 3). Subgroup analysis indicated that BMD increased with both higher doses and longer treatment durations of puerarin administered via intraperitoneal injection. The greatest increase in BMD was observed at doses ≥50 mg/kg/day, and the treatment duration was ≥8 weeks. Among the intervention methods, intraperitoneal injection showed the most favorable effect size with the lowest heterogeneity Table 2.

**TABLE 2:** Subgroup analysis of bone mineral density according to the dose, duration, and intervention method.

Subgroup	Standardized mean difference (95% confidence interval)	I2 (%)	p-value
Dose (mg/kg/d)			
≥50	3.90 [2.68, 5.13]	87	<0.00001
<50	2.40 [1.69, 3.10]	81	<0.00001
Duration (weeks)			
≥8	3.55 [2.44, 4.66]	88	<0.00001
<8	2.52 [1.84, 3.20]	76	<0.00001
Intervention method			
Gavage	3.02 [2.04, 3.99]	82	<0.00001
IP	3.92 [2.76, 5.08]	54	<0.00001
SC	2.42 [1.44, 3.40]	87	<0.00001

#### 3.4.2 Bone histomorphometric

**FIGURE 4:**
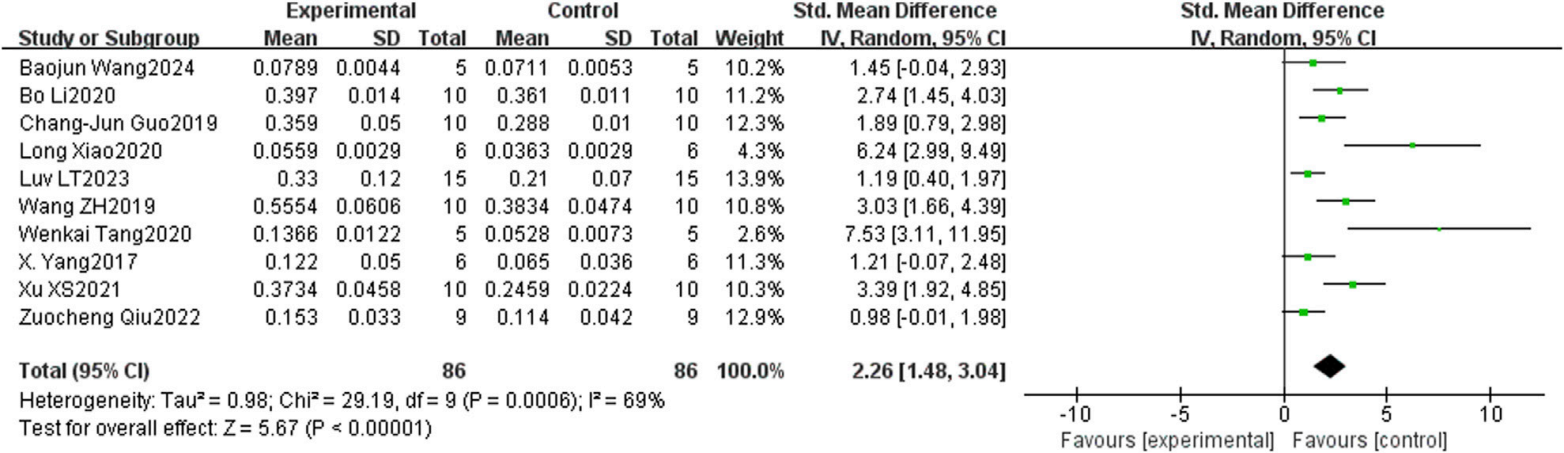
Forest plot comparing BV/TV between the puerarin group and the control group.

**FIGURE 5:**
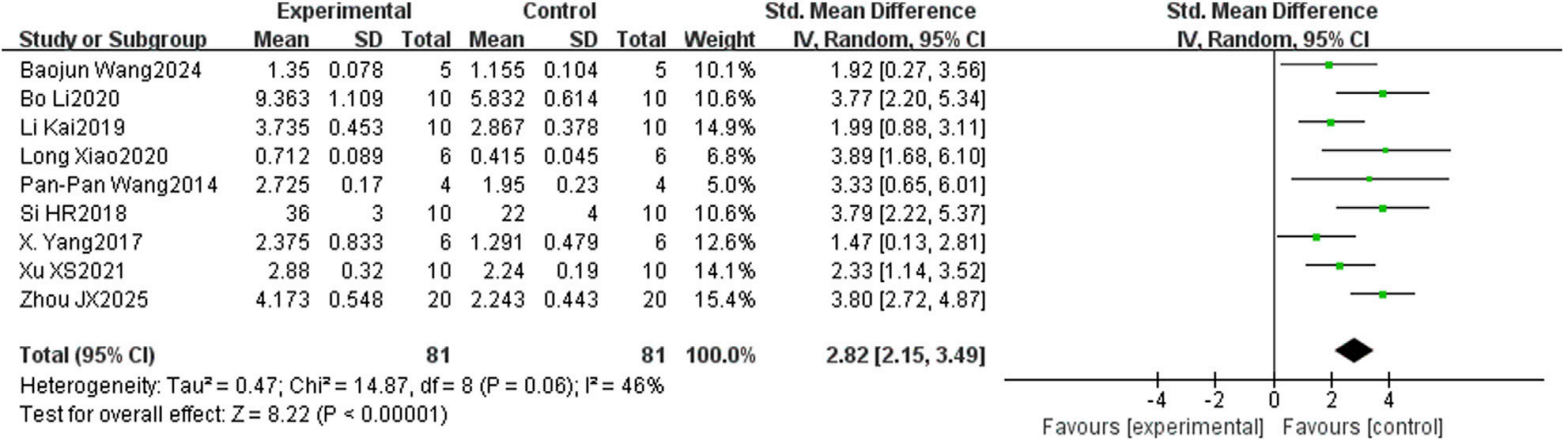
Forest plot comparing trabecular number between the puerarin group and the control group.

**FIGURE 6:**
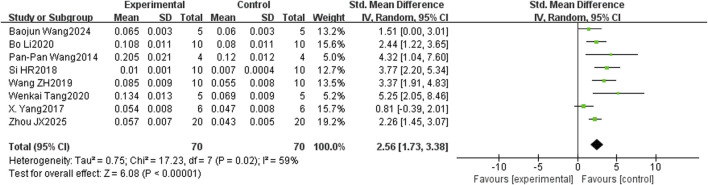
Forest plot comparing trabecular thickness between the puerarin group and the control group.

**FIGURE 7:**
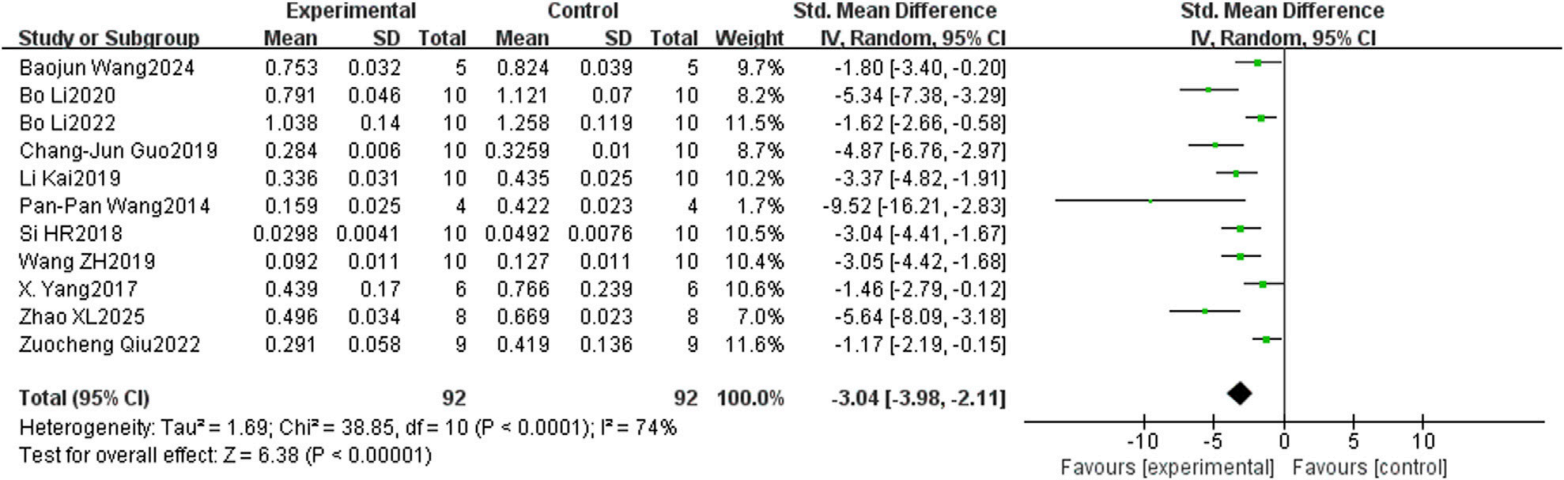
Forest plot comparing trabecular separation between the puerarin group and the control group.

In the bone histomorphometry analysis, comprising ten studies, puerarin was found to increase BV/TV (SMD = 2.26, 95% CI = 1.48 to 3.04, and p < 0.00001) (Figure 4). Nine studies reported trabecular number (SMD = 2.82, 95% CI = 2.15 to 3.49, and p < 0.00001) (Figure 5), and eight studies reported trabecular thickness (SMD = 2.56, 95% CI = 1.73 to 3.38, and p < 0.0001) (Figure 6). Additionally, eleven studies demonstrated reduced trabecular separation with puerarin treatment (SMD = −3.04, 95% CI = −3.98 to −2.11, and p < 0.00001) (Figure 7).

#### 3.4.3 Bone biochemical markers

##### 3.4.3.1 PINP

**FIGURE 8:**
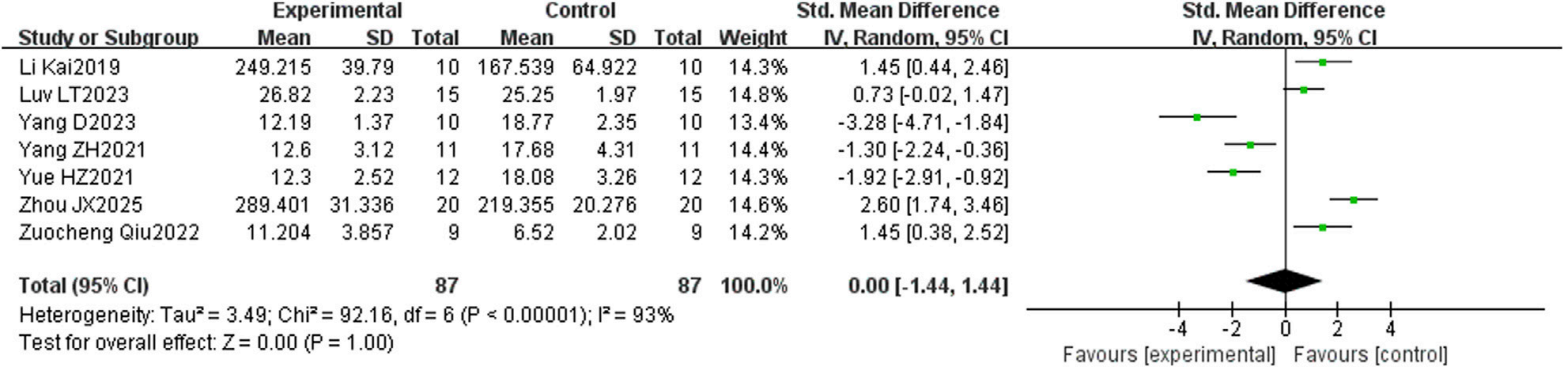
Effect of puerarin on PINP.

The meta-analysis was performed to evaluate the effect of puerarin on serum PINP levels in osteoporotic rats and was based on seven included studies. The pooled results showed no significant overall effect (SMD = 0.00; 95% CI: –1.44 to 1.44, and p = 1.00) (Figure 8).

##### 3.4.3.2 TRACP

**FIGURE 9:**
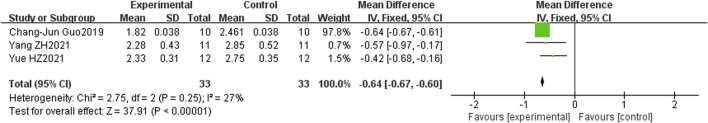
Effect of puerarin on TRACP.

The meta-analysis was conducted to assess the effect of puerarin on TRACP (tartrate-resistant acid phosphatase) levels in osteoporotic rodent models and incorporated a total of eight studies. The pooled results demonstrated a significant reduction in TRACP levels in the puerarin-treated group than in the control group (MD = −0.64, 95% CI: 0.67 to −0.60, and p < 0.00001) (Figure 9).

##### 3.4.3.3 BALP

**FIGURE 10:**
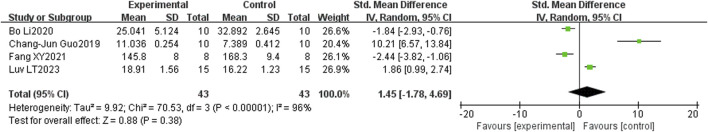
Effect of puerarin on BALP.

The meta-analysis was performed to evaluate the effect of puerarin on BALP (bone-specific alkaline phosphatase) levels and incorporated a total of four studies. The results demonstrated that puerarin had no statistically significant overall effect on BALP levels compared with the control group (SMD = 1.45, 95% CI: -1.78 to 4.69, p = 0.38) (Figure 10).

##### 3.4.3.4 CTX

**FIGURE 11:**
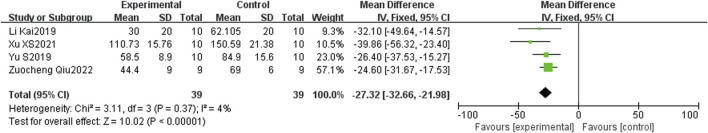
Effect of puerarin on CTX.

The findings of the meta-analysis indicated that puerarin was statistically significant in improving CTX in osteoporotic rats. CTX indicators were described in eight studies, and CTX was significantly lower in the puerarin group (MD: −27.32; 95% CI: −32.66, −21.98; and p < 0.00001) (Figure 11).

##### 3.4.3.5 OC

**FIGURE 12:**
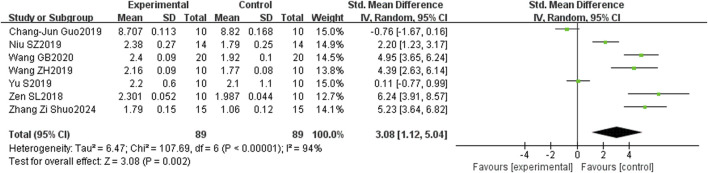
Effect of puerarin on OC.

The meta-analysis was conducted to evaluate the effect of puerarin on OC (osteocalcin) levels in osteoporotic rats and incorporated a total of six studies. The results demonstrated a statistically significant increase in the OC levels in the puerarin-treated group compared with the control group (SMD = 3.20, 95% CI: 1.12 to 5.29, and p = 0.003) (Figure 12).

##### 3.4.3.6 SCa and SP

**FIGURE 13:**
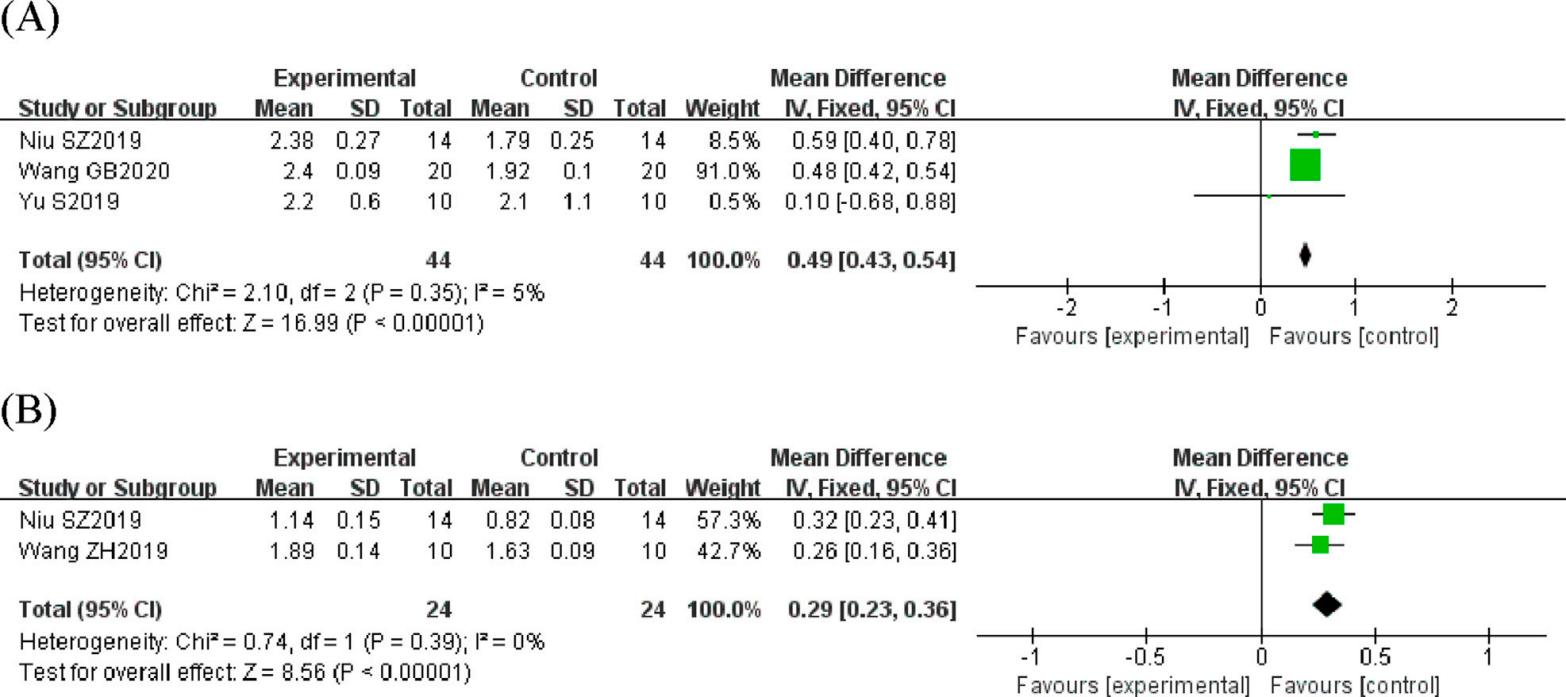
Forest plot between the puerarin group and the control group. **(A)** SCa. **(B)** SP.

Meta-analysis showed that puerarin was statistically significant in improving SCa (serum calcium) and SP (serum phosphorus) in osteoporotic rats. SCa was described in seven studies, and SP was described in six studies. Compared with the control group, the results demonstrated a statistically significant increase in SCa and SP levels in the puerarin-treated group (MD = 0.49, 95% CI: 0.43 to 0.54, and p < 0.00001) (MD = 0.29, 95% CI: 0.23 to 0.36, and p < 0.00001) (Figure 13).

### 3.5 Sensitivity analysis and publication bias

**FIGURE 14:**
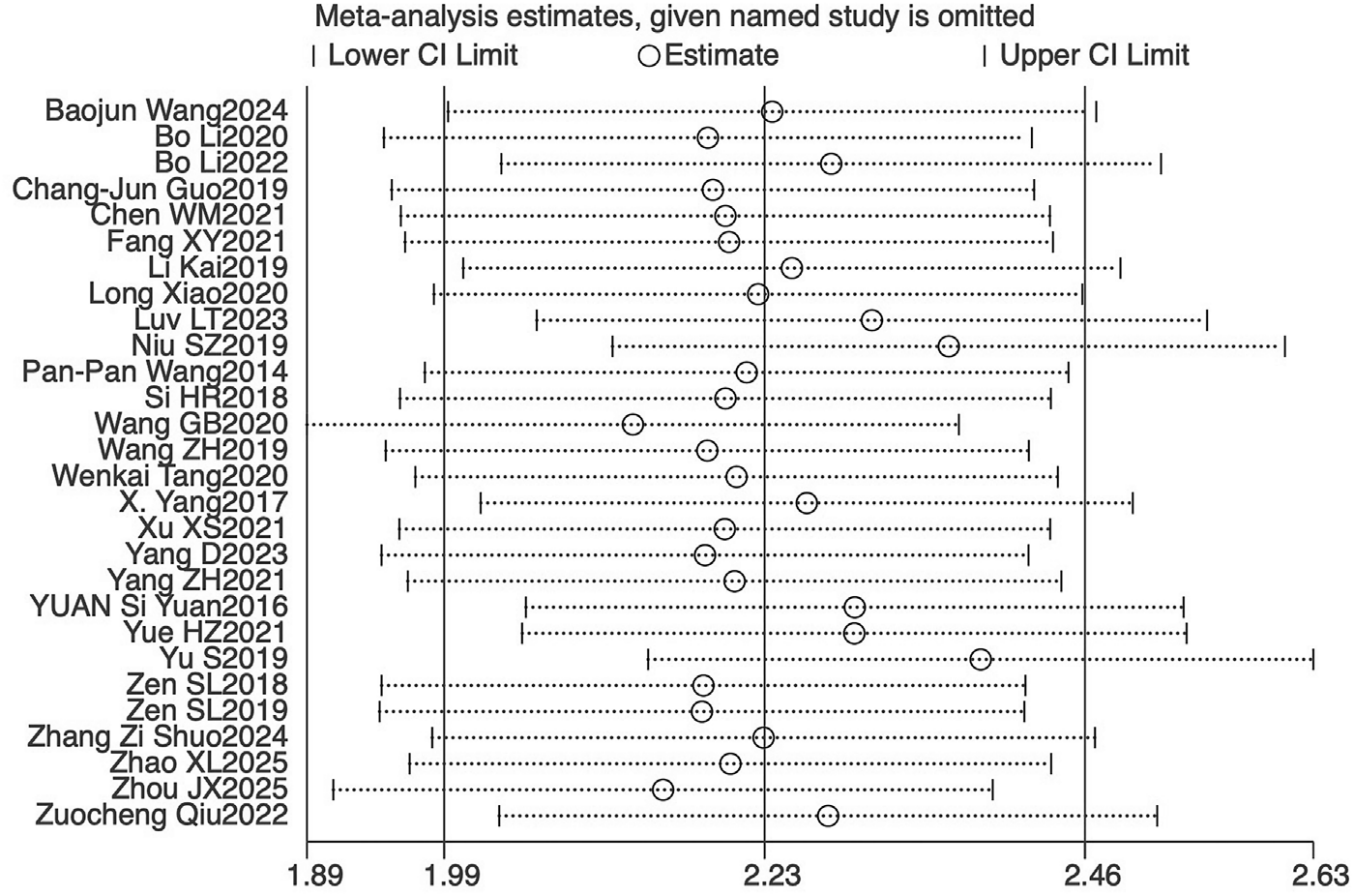
Sensitivity analysis of bone mineral density. CI, confidence interval.

**FIGURE 15:**
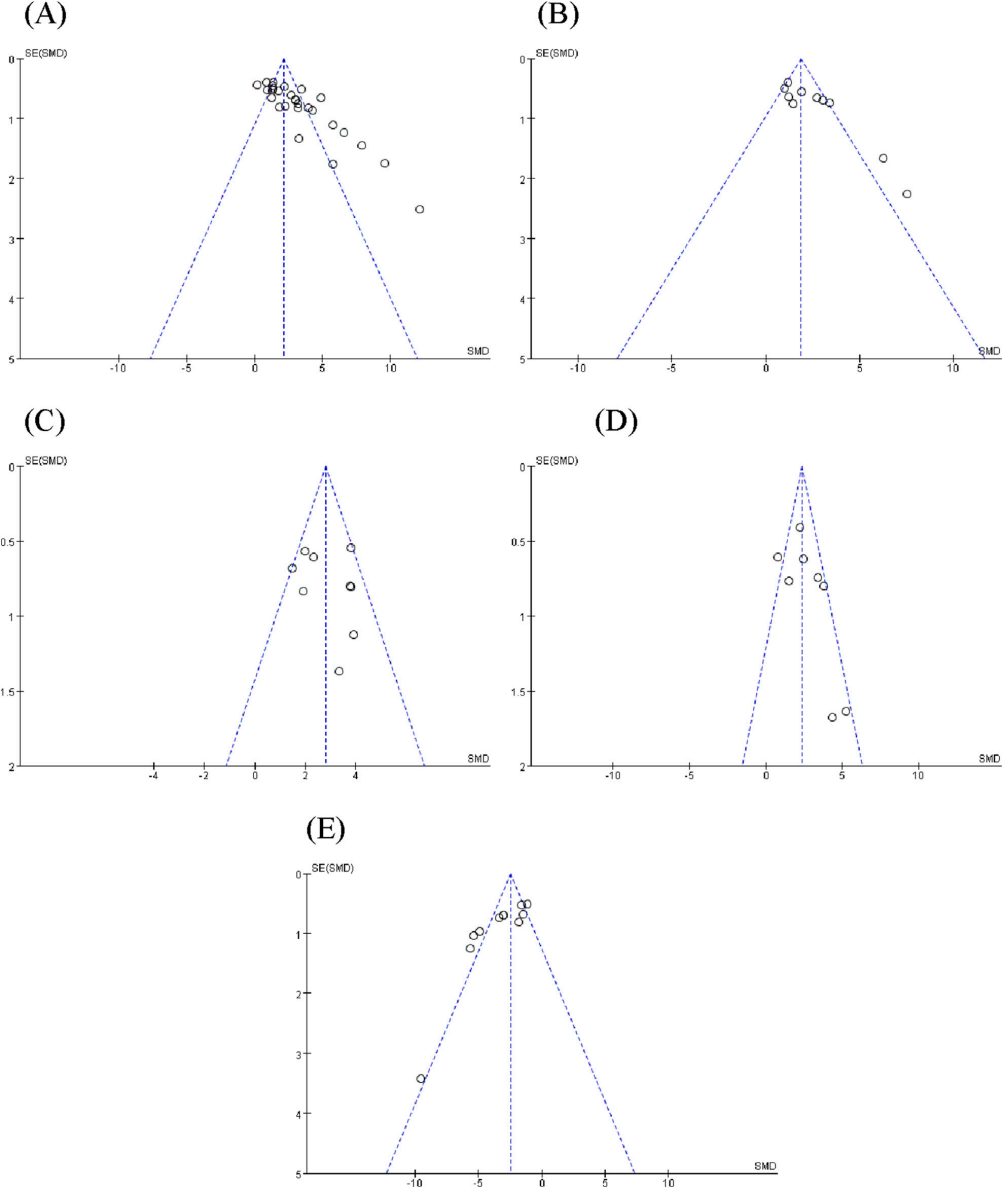
Publication bias of the effects of **(A)** bone mineral density (BMD), **(B)** bone volume fraction (BV/TV), **(C)** trabecular number (Tb.N), **(D)** trabecular thickness (Tb.Th), and **(E)** trabecular separation (Tb.Sp).

Sensitivity analysis was performed using the leave-one-out method to assess the robustness of the primary outcome (BMD). Excluding any study with no significant changes in the heterogeneity index and 95% CI indicates minimal differences among the studies, affirming the robustness of the meta-analysis results (Figure 14). To evaluate potential publication bias, funnel plot and Egger’s test were used to analyze the primary and key secondary outcomes (Figures 15A–E). Visual assessment of the funnel plots suggested a generally symmetrical distribution of studies around the pooled effect estimate for most outcomes. However, some asymmetry was observed, particularly for BMD and Tb.Sp, which indicated that there was publication bias (Figures 15A,E), and the potential publication bias might be due to the high percentage of positive results being published.

## 4 Discussion

Postmenopausal OP (PMOP) and related fractures are common clinical conditions, with elderly women facing a significantly elevated risk. Hip fractures and vertebral compression fractures, in particular, pose major health threats. Research indicates that decreased estrogen levels lead to reduced bone mass and deterioration of bone microarchitecture. The ovariectomized (OVX) rat model, which exhibits severe estrogen deficiency, is thus considered an ideal experimental subject for simulating PMOP (Black and Rosen, 2016). Consequently, this analysis specifically selected animal studies that primarily utilized the OVX modeling method. Meanwhile, we also included studies that used different OP induction models, such as glucocorticoid-induced and diabetic OP models. Although the initial induction mechanisms differ, these interventions ultimately converge on the common pathological pathways of OP, manifesting as impaired bone remodeling equilibrium that leads to reduced bone mass and deterioration of bone microarchitecture, regardless of etiology. Therefore, the inclusion of multiple validated models is necessary for achieving this broad objective.

BMD, the diagnostic gold standard for OP (Curry et al., 2018), is positively correlated with bone strength (Fonseca et al., 2014). The results demonstrated that puerarin significantly increased the BMD in osteoporotic rats. Subgroup analysis revealed that higher dosage (≥50 mg/kg/day), longer treatment duration (≥8 weeks), and intraperitoneal injection were associated with superior therapeutic efficacy of puerarin. Notably, intraperitoneal injection demonstrated a significant reduction in heterogeneity, and this finding may be attributable to the higher bioavailability and relatively stable, rapid pharmacokinetic profile of intraperitoneal administration, ensuring more predictable drug action. Although we attempted to identify sources of high heterogeneity through subgroup analyses, substantial heterogeneity persisted within most subgroups. This strongly suggests that puerarin’s efficacy is modulated by a complex network of factors, including animal strains, OP induction methods, and measurement techniques for outcome assessment. These factors may interact through complex interplay, collectively contributing to the significant variations observed across the studies.

This disease is characterized by low BMD, which signifies a reduction in the bone mass per unit volume/area and reflects a decreased amount of bone tissue and matrix (Eastell et al., 2016). Key bone histomorphometric parameters include bone volume fraction (BV/TV, representing bone mass), trabecular thickness (Tb.Th), trabecular number (Tb.N), and trabecular separation (Tb.Sp, indicating structural characteristics and is closely associated with bone volume). The present meta-analysis confirms that puerarin ameliorates these parameters, thereby exerting a therapeutic effect in osteoporotic rats. Although our findings strongly suggest that puerarin enhances bone mineral density and bone histomorphometric parameters, the absence of data on the biomechanical properties from the included studies means that its definitive efficacy in improving bone mechanical strength and reducing fracture risk requires further validation through future studies incorporating standardized biomechanical testing.

Bone remodeling consists of two complementary processes: bone formation and bone resorption, which are regulated by osteoblasts and osteoclasts, respectively (Kim et al., 2020). OP is fundamentally characterized by an imbalance in this bone remodeling process. A net loss of bone tissue occurs when bone resorption exceeds bone formation (Alippe et al., 2017), leading to the development of OP.

PINP is a specific biomarker of bone formation. Its serum level directly reflects osteoblast activity and the bone formation rate, with higher values indicating more active formation (Chen et al., 2022; Koivula et al., 2012). BALP is a classic bone formation marker, which is indicative of osteoblast activity and functional status. Elevated BALP levels, seen in conditions such as OP, Paget’s disease, and osteomalacia, suggest increased bone turnover (Bouksila et al., 2019). Although both PINP and BALP are accurate indicators of the bone formation status, the pooled effect of puerarin on these markers did not reach statistical significance in the present study. It could reflect either a genuine, inherent limitation of puerarin where its primary action is on inhibiting bone resorption rather than directly stimulating bone formation or it could stem from methodological inconsistencies across the included studies. Specifically, the high heterogeneity strongly suggests substantial variations in how these biomarkers were measured, potentially involving different assay kits, platforms (e.g., ELISA vs. RIA), and laboratory protocols. Such methodological diversity likely contributed to the inconclusive pooled result. Meanwhile, we cannot rule out the possibility that a potential stimulatory effect of puerarin on bone formation markers might require higher dosages or longer treatment periods to become apparent.

In contrast, puerarin demonstrated a significant effect in suppressing the markers of bone resorption, namely, CTX and TRACP. The serum level of CTX directly indicates osteoclast activity and the bone resorption rate; higher values correspond to more severe bone loss (Zuo et al., 2019). Similarly, TRACP activity directly reflects the number and activity of osteoclasts (Gossiel et al., 2022; Halleen et al., 2006). Furthermore, in this study, we indicated that puerarin had a positive impact on increasing the serum osteocalcin, serum calcium, and serum phosphorus levels in rodent models of OP.

The precise signaling pathways through which puerarin exerts its effects are not yet fully understood. Existing research suggests that puerarin inhibits osteoclastogenesis via the NF-κB/RANKL, TRAF6/ROS/MAPK/NF-κB, and Akt/LPS signaling pathways (Tang et al., 2020; Zhang et al., 2016). This precisely accounts for the marked decrease in CTX and TRACP levels observed in our meta-analysis. Furthermore, puerarin prevents osteoclast activation by blocking the integrin β3-Pyk2/Src/Cbl pathway (Qiu et al., 2022). It provides further evidence at the level of cytoskeletal reorganization and activation signaling for its potent ability to inhibit bone resorption. Nevertheless, the molecular mechanisms underlying puerarin’s preventive and therapeutic effects on OP require further substantiation with more robust evidence.

As a phytoestrogen, puerarin’s potential for chronic systemic exposure raises concerns about hormonal imbalance and metabolic disturbances. Although multiple studies administering high-dose puerarin (100 mg–200 mg/kg/day) for extended periods (up to 14 weeks) reported no overt signs of toxicity or mortality in the experimental animals (Li et al., 2022; Qiu et al., 2022; Zeng et al., 2019), the potential impact of its phytoestrogenic properties on other hormone-sensitive tissues requires further investigation and validation through targeted toxicological studies.

### 4.1 Strengths and limitations

The findings of this meta-analysis provide crucial preclinical evidence and design rationale for advancing the clinical research of puerarin. Given the consistent effectiveness of puerarin in suppressing the bone resorption markers (CTX and TRACP) and elevating the serum levels of osteocalcin, calcium, and phosphorus, these indicators can serve as important surrogate endpoints for evaluating its efficacy in clinical studies.

However, there are some limitations in this review. First, the methodological quality of the included studies was generally low. Most studies failed to adequately report key methodological details such as random sequence generation, allocation concealment, and implementation of blinding, which may increase the risk of selection bias, performance bias, and detection bias. The unclear or high risk of bias in these domains may lead to an overestimation of the treatment effects observed in our meta-analysis (Hooijmans et al., 2014). Additionally, a high degree of heterogeneity was observed among the studies, which we were unable to fully resolve through subgroup analyses based on the intervention duration, dosage, and intervention method. Factors with a profound influence on bone metabolism, such as baseline bone mass, precise nutritional status, physical activity levels within cages, and the estrous cycle phase in females, were rarely reported. The absence of this critical information precluded subgroup or regression analyses to account for their effects. Furthermore, combining different OP induction models, while providing a broader view of puerarin’s potential applicability, introduced biological variability and represented another source of heterogeneity. However, we were unable to further explore this heterogeneity via subgroup analysis due to the limited number of non-OVX studies. Finally, the presence of publication bias in the study results suggests that the currently included studies may have overestimated the effect of puerarin on bone mineral density. We speculate that the potential reasons for publication bias include the predominance of small-sample studies in the included literature, the existence of time-effect bias in publication trends, and the greater likelihood of positive results being published.

For future research, we recommend strict adherence to animal experiment reporting guidelines to ensure comprehensive reporting of key methodological details including randomization, allocation concealment, and blinding; standardization of modeling and evaluation criteria; enhanced implementation of blinding and randomization procedures; expansion of sample sizes and the performance of replication studies; and, in molecular mechanism research, unification of the detection methods and indicators to prevent inconsistencies in the results caused by technical variations.

## 5 Conclusion

In this meta-analysis, we demonstrate that puerarin can effectively increase bone mineral density and improve bone-related parameters in osteoporotic rats, as evidenced by changes in the biochemical bone markers, indicating its therapeutic potential. Based on the available technical data, we conclude that puerarin is a promising phytochemical capable of promoting bone density and ameliorating osteoporotic conditions. It holds promise for development into a novel alternative therapy through further large-scale experimental and clinical studies.

## Data Availability

The original contributions presented in the study are included in the article/Supplementary Material further inquiries can be directed to the corresponding author.
