# Supplementary figures and images for: The anti-osteoporotic effect of puerarin on the femoral bone in rat models of osteoporosis: a systematic review and meta-analysis
Source: Front Pharmacol. 2026 Jan 5;16:1712682. doi: 10.3389/fphar.2025.1712682 (PMC12813136; doi:10.3389/fphar.2025.1712682)

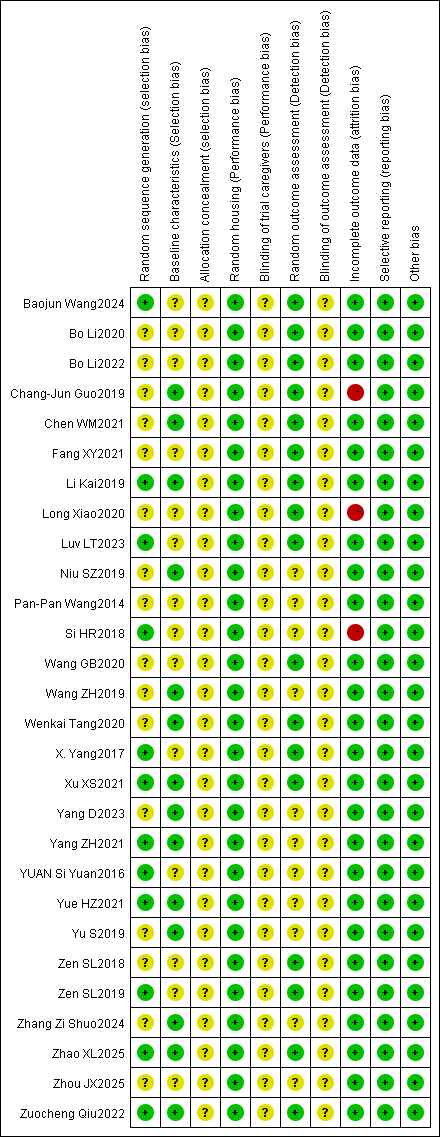

Supplement: Supplementary file 1 [file Image1.JPEG]
